# Supplementary material for: Patterns of Intron Gain and Loss in Fungi
Source: PLoS Biol. 2004 Nov 30;2(12):e422. doi: 10.1371/journal.pbio.0020422 (PMC532390; doi:10.1371/journal.pbio.0020422)
Supplement: Table S1 — Also available at http://genes.mit.edu/NielsenEtAl/. (4.3 MB ZIP). [file pbio.0020422.st001.zip › NielsenEtAl/html/47.html]

AN3583.1.NCU03219.1.MG03742.1.FG09156.1


```
 CLUSTAL W (1.82) Multiple Sequence Alignments - Introns Inserted


Sequence 1: NCU03219.1	884 aa
Sequence 2: MG03742.1	829 aa
Sequence 3: FG09156.1	843 aa
Sequence 4: AN3583.1	819 aa
Alignment Length: 911 aa
Number Identitical Residues: 317 aa
Alignment Score (without introns) 17763


MG03742.1 	MRLAYGLTLLGAASSLAAHSKVTPRDFNTNDYYVVHLASDAEPAVVARELGLTHVGQLGE
NCU03219.1	MKLSAIATPLGLAA-LAIASNVLPRDFDSNDYYVLHLDARTSPTEVARSLGLSHEGPLGE
FG09156.1 	MKIASLLSLAGLTA-LAHGSRPT-RDYDANDYYVLQLDSATTPDQVASRLGLAHEGQLGA
AN3583.1  	MRLQGAAAALGLFC-AATASLHPRRSYETRDYFALHLDASTSPSDIAARLGARHEGQVGE
          	*::    :  *  .  *  *    *.:::.**:.::* : : *  :*  **  * * :* 

MG03742.1 	LDSHHVFSGEK---APHDIVKRGLEKRGLERRSAFG-------SSALDGVLFSEKQALRS
NCU03219.1	LQDHHLFVGRK---AEHDIVKRELTERRRRKRSLGDE------PHVLDSVLFSEKQILRK
FG09156.1 	LDDHHVFRAPK---VDHDIVKREISERKRRKRDLGG-------SDPVDGILLSKKQQARQ
AN3583.1  	LDGHHTFSLPRENGADLDALLEDLQIKRKLRRRDGDLGALDKRDKDLAGILWSQKLTPKQ
          	*:.** *   :....  * : . :  :   :*   . .: ..    : .:* *:*   :.

MG03742.1 	PWPKRVPPPPRAGPSPFRARQEDAPDANAVKKLTQIAKTLNIEDPIFHEQWHLFNSVQLG
NCU03219.1	PWEKRMVPRP-VGPS-RRGLFSDQEVDWAVKKQNEVIGNLGIRDPIFKEQWHLFNTVQTG
FG09156.1 	HLFKRDVPLNQRRWKPFSSR-ADAKVAELAEYQETIMKQLEIQDPIFKEQWHLLNPLQPG
AN3583.1  	RLVKRTPPPEKLARDPSSDG-EEKEDVEAAQARSKLVSTLGIEDPIFTKQWHLFNTVQVG
          	   **  *      ..      :      .:    :   * *.**** :****:*.:* *

MG03742.1 	HDVNVTDVWLSGVTGKNATVSIVDDGLDMYSDDLKGNYYAKGSYDFNDKTEEPKPRLSDD
NCU03219.1	HDVNVTGLWLEGVTGKNATVAIVDDGLDMETDDLKDNYYAQGSWDFNDKGPDPKPRLSDD
FG09156.1 	HDVNVTGLWLEGITGKNVTVAVVDDGLDMNSDDLKPNYFAEGSWDFNDNDPEPAPVLDDD
AN3583.1  	HDLNVTGVWLEGITGKGATAAIVDDGLDMYSNDLSPNYFPEGSWDFNDHTAEPRPRLRDD
          	**:***.:**.*:***..*.::******* ::**. **:.:**:****:  :* * * **

MG03742.1 	RHGTRCAGEVAAVRNDVCGVGVAFDAKVSGLRILSKLISDADEAVALNYDFHNNHIYSCS
NCU03219.1	KHGTRCAGEVSAGKNKACGVGVAYDSRIAGLRILSKLISDADEAVAMNYDFQHNQIYSCS
FG09156.1 	RHGTRCAGEVAAARNDVCGVGVAYDSKVAGIRILSKLISDADEAEALMYKYHDNHIYSCS
AN3583.1  	RHGTRCAGEVAAARNDVCGVGVAYDSRIAGIRILSGPIDDTDEASAINYAYQENDIYSCS
          	:*********:* :*..******:*::::*:****  *.*:*** *: * ::.*.*****

MG03742.1 	WGPPDDGKSMDAPGLLIRRAMLNAVQKGRDGKGSIYVFASGNGAANEDNCNFDGYTNSIY
NCU03219.1	WGPPDDGQSMDAPGILIKRAMLNAVQKGRGGLGSIYVFASGNGAGNGDNCNFDGYTNSIY
FG09156.1 	WGPSDDGQTMEAPDVVIRRAMLKAIQEGRSGLGSVYVFASGNGAGQGDNCNFDGYTNSIY
AN3583.1  	WGPPDDGATMDAPGILVSRAIVNGVQKGRDGKGSIFVFAAGNGAASGDNCNFDGYTNSIY
          	***.*** :*:**.::: **:::.:*:**.* **::***:****.. *************

MG03742.1 	SITVGAIDRKGLHPYYSEACSANLVVTYSSGSGDAI0HTTDVGTNQCYNGHGGTSAAAPL
NCU03219.1	SITVGAVDRNGDHPYYSESCSANLVVTYSSGGGDSI~HTTDVG-NACSDTHGGTSAAAPL
FG09156.1 	SITVGAVDRTGLHPYYSEECSAQLVVTYSSGSGDAI0HTTDVGKNSCYKAHGGTSAAAPL
AN3583.1  	SITVGAIDREGQHPQYSESCSAQLVVAYSSGISDAI~STTDVGTDRCYSVHGGTSAAGPL
          	******:** * ** *** ***:***:**** .*:*  *****.: * . *******.**

MG03742.1 	AAGIFALVLEVRPDLTWRDMQYLAFMTAVPLNLDSGDWQDTTIGKKYSHTYGYGKVDTFG
NCU03219.1	AAGIFALVLQVRPDLSWRDMQYLTVNTAVPINLDSGEWQTTAIGKQFSHMYGYGKLDSYA
FG09156.1 	AAGIFALVLQVRPDLTWRDLQYLAMDTALPIEGDEANQQNTTIGKKFSHTFGYGKIDSWA
AN3583.1  	VVGAISLALSVRPELTWRDAQYIVLETAVPVHEDDGSWQVTKSGRKFSHDWGYGKIDVYS
          	..* ::*.*.***:*:*** **:.. **:*:. *... * *  *:::** :****:* :.

MG03742.1 	IVEAAKTWKLVKPQAYFFSPWIHVKHGIPEGDAGLATKFE0STLAVA-------------
NCU03219.1	IVQAAKTWKKVKPQAWFYSPWIHVNKAIPQGDTGVAVSYE~VTQAMLDEANVERLEHITV
FG09156.1 	LVEKAKDWKLVKPQSWYFSPWIHVKKAIPEGRDGLTVTLD~VTEDMLKDSNLARVEHVTV
AN3583.1  	LVQKAKTWELVKPQAWYHSPWLRVQHEVPQGDKGVAASWE~VTEQMMKDANLEKLEHVTV
          	:*: ** *: ****:::.***::*:: :*:*  *::.. :  *  : ..:.    .  : 

MG03742.1 	----------DLSVDLISPNKVISHLSVTRRLDNAKSGYKDWTFMSVVHW~GESGVGTWT
NCU03219.1	TMNIMHTRRGDLSVDLISPNNLVSHLSVSRKNDEARAGYDDWTFMSVVHW2GETGVGNWT
FG09156.1 	TMNVEHTRRGDLSVDLISPDNVVSHLAVSRRSDAKDAGYVDWTFMSVAHW2GESGVGKWT
AN3583.1  	TMNVNHTRRGDLSVELRSPEGIVSHLSTPRKNDNAEVGYIDWTFMTVAHW2GESGVGTWT
          	: .   :  .****:* **: ::***:..*: *    ** *****:*.** **:***.**

MG03742.1 	IVVRDTKANEFQGNFTDWHLKLWGEARDASKAVLLPMPTENDDKDHDKPS-TTTVAAETI
NCU03219.1	IIVKDTQINSHEGVFTDWHLKLWGESKDASKAKVLPMPTEEDDNDHAAIP-TTTLPATTT
FG09156.1 	IIVRDTEKNSYKGKFTDWRMKLWGEAIDADKATLLPMPNPGDDDDHDKIVSTTTIAASTT
AN3583.1  	VIVKDSTVNDNVGEFIDWRLNLWGMAVDGAKQKLHPLPDEHDDDHS-----IEDAIVVTT
          	::*:*:  *.  * * **:::*** : *. *  : *:*   **..           . * 

MG03742.1 	TMPPGSASTPDKPITTPSSGHPDRPVN-NKPTGAGSPGSQVSQVTTDQGSAVSGSPATQS
NCU03219.1	TLPPQPIATDTKPSTVS--DHPNRPVN-SKPTPTDAPNP--SPTSDEQTSETT----TPA
FG09156.1 	TAPP---ATKPTLIDHP-SDHPERPTKPARPSGTEEEESSSTQTSAEETSTAS-------
AN3583.1  	SVQPAPTATEAPVEPSH---TVDRPVK-VKPTSTGSASETETPTPTPTPASDS-------
          	:  * . :*             :**.:  :*: :       : ..    :  :       

MG03742.1 	NSSWLPSFLPTFGVSSHTQVWIYGALGLIVVFCIGLGIYLFMARQRRLRNSPRDNYEFEL
NCU03219.1	NSTWLPSFLPTFGKSAATMVWVYGSAALIGVFCISLAVYFWLARRKRLRNNPRGDYEFEL
FG09156.1 	-SSWV-SWLPTFGASKKAQIWIYGAVGFIAAFCVGLGVYFWIARRRRLRNSSHNSYEFEL
AN3583.1  	---FLPSFFPTFGASKHTQVWIYAAISSIIVFCIGLGIYFHVQRRRRLRND-RDDYDFEM
          	   ::.*::**** *  : :*:*.: . * .**:.*.:*: : *::****. :..*:**:

MG03742.1 	LDEEEAEGLNRG----EK---GFTGGQKGRKTRGGELYDAFAGGSDDEDE----------
NCU03219.1	LDEEEAEGLAGGGSSAEKRRAGAGGLAGGKRTRGGELYDAFAGGSDDEDADDGVYRDRVD
FG09156.1 	IDEEEAP-LNAG----EK-----GPGNKPRRTRGGELYDAFAEGSDEEPF----------
AN3583.1  	IEDEDEAKAMNG----------------SSRRRGGELYNAFAGESEEEPL----------
          	:::*:      *                  : ******:***  *::*            

MG03742.1 	---FATEQYHDRAEGS---------------AERTHDNDDDGHHVIGDDSEDE--DEK--
NCU03219.1	EVGSGSGSGSERASGSGSGSGSAGNGGRQARALRSDSDDESGHHVVGDDDDDD--DESDN
FG09156.1 	------EDYRDNREPS----------------RDRLAGDDTQHYAVGDDSDDSESDDDSS
AN3583.1  	--------FSDEDDDE-------------------PYRDQTTGAELRTSADDTRRND---
          	          :. . .                      *:     :  . :*   ::   

MG03742.1 	-------RPMTSR-
NCU03219.1	DDGGHGSRPLRR--
FG09156.1 	DGARAETRPLGGRR
AN3583.1  	--------------
          	
```
